# Perceived social contribution and its associations with political participation

**DOI:** 10.1371/journal.pone.0330385

**Published:** 2025-09-05

**Authors:** Ellen C. Reinhart, Monique M. Turner, David M. Markowitz, Dustin Carnahan

**Affiliations:** Department of Communication, Michigan State University, East Lansing, Michigan, United States of America; National Cheng Kung University, TAIWAN

## Abstract

Many people who are eligible to participate in the political process do not, suggesting the interests of a large portion of the electorate are not adequately represented in government. While some past work has found that subjective well-being is related to political engagement, less is known about which specific aspects of well-being might drive this effect. We propose and test the idea that self-perceived social contribution – the belief that one’s life and everyday activities provide something of value to society – is related to multiple forms of political participation, likely because people who believe they provide something of value to society feel more integrated with society and therefore may be more likely to act on its behalf via political participation. Two correlational studies (N = 3,729) with data from distinct points in American politics (1996 and 2024) find that individuals with greater self-perceived social contribution were more likely to intend to vote, be willing to engage in activism, seek rather than avoid election information (Study 1), and donate to and volunteer for political causes (Study 2). Further, Study 2 provides empirical support for the previously theorized components of social contribution, providing evidence that self-efficacy and social responsibility underlie this construct in political contexts. Together, these studies identify a specific dimension of well-being that is related to multiple forms of political participation and suggests that fostering feelings of social contribution may promote democratic engagement.

## Introduction

Political participation is essential to a representative democracy, yet a significant number of eligible citizens do not engage in fundamental democratic processes. For example, due to several reasons ranging from structural (e.g., access to polling places and transportation) to individual (e.g., dissatisfaction with the candidates and political system), many people who are eligible to vote do not [1–3]. In the most recent national election in the United States in 2024, 64% of those eligible voted on Election Day, with nearly 90 million eligible voters who did not vote [4,5]. While this voter turnout rate is relatively high compared to historical U.S. voter trends (with the exception of 2020 in which 66% of the vote share turned out to vote), it still lags many comparable advanced democracies with much higher voter turnout rates [6]. Further, voting turnout is often patterned by social class, race and ethnicity, gender, and their intersections, suggesting some of the most consequential political contests are determined by those who are not representative of the larger voting eligible population [7]. This is a critical problem because people who do not vote are less likely to have their interests represented by the officials elected to represent them [8].

Social scientists have long studied political participation, with a great deal of focus paid to the role of institutional factors (e.g., structure of the political party system), social networks and social capital, as well as demographic characteristics (e.g., education, age, gender, and race and ethnicity) [9–12]. A growing literature has found that people with high (versus low) levels of reported well-being, who are satisfied with their lives, and who do not experience depressive symptoms are more likely to engage with the political process through voting, volunteering, and activism [13–17]. Though, several other studies find little or no evidence for the relationship between well-being and political participation, suggesting the link is complex and likely boundaried or context-dependent [18–21]. Further, much less is known about the specific aspects of well-being that might impact political participation. Here, we extend the literature on well-being and political engagement by investigating if self-perceived social contribution, or how much one thinks their life and everyday activities provide something of value to society [22], relates to political participation above and beyond more global measures of well-being. Given that a sense of contributing to society is associated with feeling a part of the larger collective and integrated within it, we expect that perceived social contribution will be associated with a desire to be further involved in society via greater involvement in the political system.

### Well-being and political participation

While some research has focused on the potential well-being benefits (e.g., greater sense of meaning) of engaging in the political system (e.g., political volunteering, activism) [17,23,24], a growing literature has investigated the impact of subjective well-being on political participation [13,14,18]. Some scholars have speculated that a sense of satisfaction with one’s life might lead to political apathy if the political system is not needed for personal gain [25]. However, other work has noted that happiness and well-being are generally associated with a host of positive, prosocial behaviors [26,27]. Further, some research has drawn parallels to the sociological theory of post-materialism [e.g., 28,29], which suggests that achieving one’s desired satisfaction in life positions people to look beyond their own immediate needs to address broader societal and collective questions and challenges. However, postmaterialist values are theorized and shown to be most consistently related to non-conventional forms of political engagement, including protesting and boycotts [29,30].

Several correlational studies suggest people who are happier, more satisfied with their life, and report lower levels of depression are more likely to vote and donate to and volunteer for political causes [14–16,31]. Further, subjective well-being is linked mostly with “non-conflictual” forms of political participation (e.g., voting, donating to political campaigns) but not forms of political participation that are more likely to involve conflict, such as attending a protest [14]. Some work has also suggested that voters’ subjective well-being impacts who they vote for, with people who are relatively more satisfied with their life being more likely to vote for incumbents and people relatively less satisfied with their life being more likely to vote for populists [32–35]. While this literature has identified some links between subjective well-being and political engagement, it includes many contradictory findings and provides little insight into what aspects of well-being may contribute to these relationships. Against this backdrop, more empirical work is needed to understand the mechanisms potentially driving any relationship between subjective well-being and political participation. Here, we propose and test whether one’s self-perceived social contribution is particularly important for political participation.

### Perceived social contribution

Social contribution reflects the belief that whatever you do in the world is valuable and is recognized as such by others [22,36]. Theorized as a part of social well-being [22], self-perceived social contribution can serve as a measure of one’s perceived social value in society and reflects both the belief that one’s actions contribute to society and that others in the broader society agree one’s actions and life are valuable. Notably, this construct does not define what a contribution is or what best constitutes a meaningful contribution. In other words, it focuses on the *perception* that one contributes, not the specific actions one might take to contribute to society. Keyes’ original conceptualization of social contribution posits that the construct “resembles the concepts of efficacy and responsibility” [22]. Self-efficacy refers to the belief that one can execute certain behaviors to achieve their goals [37], while a sense of social responsibility corresponds to the obligation one feels to positively impact society [38]. Based on this conceptualization, having the ability to produce intended results and an obligation to focus on what is owed to others and society both relate to feeling as though one contributes to society. Together, social contribution reflects the belief that one is capable of achieving their goals and the desire or obligation to provide something of value to a larger collective.

Feeling as though one provides something of value to society is related to a host of positive health and well-being outcomes. As one example, perceived contribution serves as a buffer against the negative impacts of chronic pain on subjective well-being through perceived social support [39]. That is, midlife and older adults who reported greater perceived social contribution maintained better social support networks despite their chronic pain and as a result experienced better psychological well-being outcomes (e.g., their sense of autonomy, personal growth, etc.). Further, people who report higher levels of perceived contribution are more likely to live longer even when controlling for baseline health measures [40]. While the benefits of believing you contribute to society have been well-documented on health and well-being, less is known about how this perception may extend to other positive outcomes, such as the health of a politically engaged society.

Here, we theorize that feeling as though one makes a meaningful contribution to society will relate to positive political participation, as people who view themselves as contributors are more likely to identify with and feel integrated within that broader collective [22,41]. Thus, we suggest that enhanced integration within society may motivate future action, involvement, and investment in society via political participation [12,42,43]. On the other hand, feeling as though one does not provide something of value to society likely corresponds with a sense of disintegration with society and alienation, which other work suggests are often associated with reduced political participation [44,45]. Additionally, self-efficacy and social responsibility that resemble a sense of contributing likely both independently galvanize action and motivate behaviors to impact society more broadly. Thus, we argue that self-efficacy and social responsibility should be linked to perceived social contribution, which in turn is related to political engagement. We are unaware of prior work that has tested these links directly and thus, we also provide one of the first examinations understanding how perceived social contribution is supported by self-efficacy and social responsibility within the context of political participation.

### The current paper

In two studies summarized in Table 1, we investigated the degree to which self-perceived social contribution is related to measures of political participation, including intending to vote, willingness to engage in activism, seeking and not avoiding information about an election (Study 1), and average donations to and volunteering for political causes (Study 2). In Study 2, we also investigated if self-efficacy and social responsibility, as originally theorized, are indeed related to self-perceived social contribution within the context of modeling political outcomes. We specifically test the following research questions across two studies including data collected in 1996 and 2024 (total *N* = 3,729 across studies). The first study examined these research questions in the context of the 2024 U.S. Presidential election. Upon finding results that support the link between perceived social contribution and political participation (RQ_1_), we replicated these results with the publicly available Midlife in the United Statues (MIDUS) dataset of adults for generalizability purposes while controlling for more global measures of well-being and examined how self-efficacy and social responsibility are associated with self-perceived social contribution (RQ_2_):

**Table 1:** Summary table of analyzed studies.

	Study 1	Study 2
Sample source	Emotion Election Dataset	MIDUS Wave 1
Year	2024	1996
N	1,049	2,680
Research question addressed	RQ_1_	RQ_1_ and RQ_2_
Political participation outcomes	Voting intention, willingness to engage in social activism, information seeking, information avoidance	Donating to political causes, volunteering for political causes
Robustness check controls	Political efficacy	Life satisfaction, psychological well-being

RQ_1_: Does believing one contributes to society relate to political participation?

RQ_2_: Are self-efficacy and social responsibility related to self-perceived social contribution?

## Study 1: Contribution and voting intention, activism, and information seeking in the 2024 election

### Method

#### Participants.

Adults living in the U.S. completed an online survey between September 30 – November 25, 2024. The data were collected via a survey panel company who recruited a sample that was roughly representative by race and ethnicity (66% participants who are non-Hispanic and White, 15% participants who are non-Hispanic and Black, 12% participants who are Hispanic, and 10% participants who report another race and ethnicity), sex (50% male, 50% female), and political orientation (49% Democrat, 48% Republican, 3% Independent). Approximately 200 participants were surveyed per week, spread out among the different days of the week. Because we were interested in the relationship between how one feels about their contributions to society and political participation in the future, we excluded participants who took the survey on or after Election Day (November 5, 2024). The final sample (N = 1,049) was roughly balanced by the two major political parties in the U.S. (48.4% Democrat, 46.8% Republican, 3.2% Independent, < 1% Other or Prefer not to say). See Supplemental Information (SI) for full sample demographics. The Michigan State University Institutional Review Board approved the study prior to data collection (protocol: 11252). At the start of the survey, participants read about the study and agreed to voluntarily participate by selecting “agree,” which served as the informed consent in lieu of written or typed documentation per the ethics committee standards. Study materials, anonymized data, and analysis code for both studies can be found on the Open Science Framework: https://osf.io/sdxzu/

#### Measures.

Participants completed an online survey that included various measures relating to attitudes towards the 2024 Presidential election as well as their sense of contributing.

**Self-perceived social contribution.** Participants indicated how much they agreed with each of three statements from 1 *strongly disagree* to 7 *strongly agree*: “I have something valuable to give to the world,” “My daily activities do not create anything worthwhile for my community” (reverse scored), and “I have nothing important to contribute to society” (reverse scored). The scale is the mean of the three items (*M* = 4.94, *SD* = 1.40, α = .71).

**Voting intention.** Participants indicated if they intended to vote in the 2024 Presidential Election. Participants selected “yes,” “no,” “I don’t know,” or “not applicable.” Only participants who selected “yes” or “no” were included in the analysis, with the vast majority of participants (95.3%) intending to vote.

**Willingness to engage in social activism.** Participants responded to the following prompt for each of 13 actions: “With regard to this presidential election, I would be willing to…” from 0 = *0% willing to do this action* to 100 = *100% willing to do this action*. The actions included posting about a candidate on social media, attending a protest, and volunteering for a candidate or organization, among other actions (see S1 Appendix for full list). Following the protocols set by the scale-developers, these actions were then weighted in terms of the difficulty and level of commitment required [46]. The scale-developers determined the weights for specific actions by asking participants to rate the level of commitment required to engage in each action and used the median of the responses to determine the weight for the action. Such an approach recognizes that activist behaviors are escalating in nature and should not be treated as comparable (e.g., putting a sign in one’s yard is considerably less difficult and requires less effort than organizing a protest). For example, the action “post about my candidate on social media” was multiplied by 59 whereas a more labor-intensive “volunteer for my candidate’s campaign” was multiplied by 70 (see S1 Appendix for full weights). The scale is the z-score of the sum of the 13 weighted responses.

**Information seeking versus avoidance.** Participants indicated how much they agreed with one statement about their *information seeking* (“I plan to seek information about the 2024 presidential election in the near future,” **M* *= 3.84, *SD* = 1.17) and one statement about their *information avoidance* (“I avoid information about the 2024 presidential election”; **M* *= 2.13, *SD* = 1.27) on a scale from 1 *strongly disagree* to 5 *strongly agree*. Because past work suggests information seeking and avoidance are separate processes that are motivated by distinct mechanisms, we did not combine these two items into a scale [47].

**Covariates.** Because prior work has shown self-perceived social contribution and political participation can vary by demographic markers, including social class, age, gender, race and ethnicity, marital status, and working status [3,36,48,49], we included measures of these markers as covariates. Social class (as operationalized by the highest level of education received and dichotomized into participants with or without a four-year college degree), age, gender, race and ethnicity (dichotomized into participants who are White and Participants of Color), marital status (dichotomized into married and not married), and working status (dichotomized into currently working and not currently working) were included as covariates in all analyses. To account for any role participant political identity may play, party identification (dichotomized into Republican or Democrat with participants who selected “Other” or “Independent” being asked to indicate if they leaned more towards the Democratic Party or Republican Party and coded as such) was also included as a covariate. See S1 Appendix for full covariates descriptives.

### Results

For both studies, we used R (version 4.4.1), including *tidyverse* (version 2.0.0) to wrangle and visualize data and *rcompanion* (version 2.5.0) to estimate pseudo R-squared for generalized linear models [50–52]. In Study 1, 95% CI represent 5000 bootstrapped confidence intervals. Nagelkerke pseudo R-squared is reported for models with binomial distributions. All analyses are robust to dropping covariates. See S1 Appendix for analyses without covariates.

#### Voting intention.

We conducted a logistic regression with self-perceived social contribution as the independent variable and the likelihood of intending to vote versus not intending to vote as the dependent variable while controlling for the covariates.

Self-perceived social contribution was significantly related to a greater likelihood of intending to vote in the 2024 Presidential election (*B* = 0.46, *SE* = 0.12, 95% CI [0.22, 0.71]; *z* = 3.73, *p* < .001, *R*^2^ = .34).

#### Willingness to engage in social activism.

A multiple regression revealed that self-perceived social contribution was significantly positively related to a willingness to engage in social activism (*B* = 0.10, *SE* = 0.02, 95% CI [0.05, 0.14]; *t* = 4.27, *p* < .001, *R*^2^ = .05).

#### Information seeking versus avoidance.

Two multiple regressions showed that self-perceived social contribution was significantly positively related to seeking more information about the 2024 election (*B* = 0.16, *SE* = 0.03, 95% CI [0.10, 0.21]; *t* = 5.89, *p* < .001, *R*^2^ = .04) and significantly negatively related to avoiding information about the election (*B* = −0.27, *SE* = 0.03, 95% CI [−0.32, −0.22]; *t* = −9.97, *p* < .001, *R*^2^ = .17).

#### Robustness check.

Given that some past work has controlled for internal and external political efficacy when modeling political participation [14], we conducted robustness checks controlling for internal and external political efficacy to address whether social contribution’s effects operate directly or indirectly through political efficacy. As shown in S1 Appendix, the pattern of results remained largely consistent when controlling for political efficacy. Specifically, self-perceived social contribution was significantly associated with voting intention, information seeking, and information avoidance. In contrast, the association between self-perceived social contribution and willingness to engage in social activism was positive but failed to reach significance once the two measures of political efficacy were included in the model. This pattern suggests that activism, compared to other measures of political participation, may be more closely tied to political efficacy more broadly. Overall, with three of the four outcomes remaining significant when controlling for internal and external political efficacy, these results suggest that self-perceived social contribution is associated with political participation above and beyond measures of political efficacy.

### Discussion

Study 1 found that in a recent political contest, the 2024 U.S. Presidential election, self-perceived social contribution was significantly related to multiple measures of political participation. Specifically, individuals who reported greater levels of contribution were more likely to intend to vote, be willing to engage in social activism, and seek rather than avoid information about the election. It should be noted that the relationship with willingness to engage in social activism was not robust to controlling for political efficacy, suggesting that efficacy beliefs may partially mediate or confound this particular relationship. Study 2 builds on these findings by investigating whether the relationship between self-perceived contribution and political participation replicates in another sample of adults from 1996 and whether it remains robust when controlling for global measures of well-being. Additionally, Study 2 investigates if the theoretical components of self-perceived social contribution (i.e., self-efficacy and social responsibility) underlie the construct in the political context.

## Study 2: Contribution and political donations and volunteering in MIDUS (1996)

### Method

#### Participants.

Study 2 included the random-digit-dialing subsample of the Midlife Development in the United States (MIDUS) national survey. Though MIDUS has multiple waves, the social responsibility measure was only included at Time 1, so we only include data from the first wave of data collection which was administered from 1995–1996. The data were accessed on March 18, 2018. All MIDUS data collection included informed consent and was approved by respective institutional IRB committees. The authors did not have access to any information that could identify participants from this dataset. The sample was restricted to only include participants who responded to all the measures relevant to Study 2. The final sample (N = 2,680) was roughly balanced by gender (50.71% women, 49.29% men), predominately White (85.58%; 11.42% Participants of Color), majority without a four-year college degree (69.48%; 30.52% with a four-year college degree), and with age spanning 20–74 years (*M*_Age_ = 46.61 years, *SD*_Age_ = 12.92). See S1 Appendix for full sample demographics.

#### Measures.

***Self-perceived social contribution scale.*** Self-perceived social contribution was measured using the same 3-item scale used in Study 1 (*M* = 5.17, *SD* = 1.27, α = .68).

#### Components of self-perceived social contribution.

***Self-efficacy.*** Participants indicated how much they agreed with each of four statements from 1 *strongly disagree* to 7 *strongly agree*: “I can do just about anything I really set my mind to,” “When I really want to do something, I usually find a way to succeed at it,” “Whether or not I am able to get what I want is in my own hands,” and “What happens to me in the future mostly depends on me.” The scale is the mean of the four items (*M* = 5.85, *SD* = 1.01, α = .70).

***Social responsibility.*** Participants indicated how obligated they would feel in four hypothetical situations on a scale from 0 *no obligation at all* to 10 *a very great obligation*: “To serve on a jury if called,” “To keep fully informed about national news and public issues,” “To testify in court about an accident you witnessed,” and “To vote in local and national elections.” The scale is the mean of the four items (*M* = 7.69, *SD* = 1.94, α = .78). Given that one of the items asks specifically about voting which may influence the relationship between social responsibility and political participation due to its political nature, we conducted the same analyses without this item in the scale. The results are reported in S1 Appendix and show a consistent pattern of results regardless of whether the scale includes the voting item.

#### Political participation.

***Donating to political causes.*** Participants indicated how much money, if any, they donate to political causes on average per month. Because the vast majority (84.03%) of the sample did not donate to a political organization monthly, we dichotomized this variable into donated to political causes (15.97%) or not.

***Volunteering for political causes.*** Participants indicated how many hours, if any, they volunteer for political causes on average per month. Because the vast majority (90.60%) of the sample did not volunteer for a political organization monthly, we also dichotomized this variable into volunteering for political causes (9.40%) or not.

#### Covariates.

***Demographics.*** The same demographic covariates (e.g., age, gender, race and ethnicity) that were included in Study 1 were included here. Political party identification was not measured in this dataset and therefore not included as a covariate.

***Global well-being variables.*** To account for global levels of well-being, we included a single-item measure of *life satisfaction* (“How would you rate your overall life these days?” measured from 0 *the worst possible life overall* to 10 *the best possible life overall*; **M* *= 7.65, *SD* = 1.66; [53]) and an 18-item measure of *psychological well-being*, including items assessing positive relations with others, self-acceptance, autonomy, personal growth, environmental mastery, and purpose in life, measured from 1 *strongly disagree* to 7 *strongly agree*; *M* = 5.53, *SD* = 0.79, α = .82 [54]) as covariates.

### Results

To understand the relationship between self-perceived social contribution, political participation outcomes (i.e., donating to and volunteering for political causes), and the theorized components of social contribution (i.e., self-efficacy and social responsibility), we conducted a structural equation model (SEM) using the *lavaan* package (version 0.6.19) [55]. The parallel mediation model specified self-efficacy and social responsibility as additive predictors of political participation (i.e., donations and volunteering; two binary variables) as mediated by self-perceived social contribution, examining four indirect pathways. The model controlled for demographic covariates and measures of global well-being (i.e., life satisfaction and psychological well-being). The model used weighted least squares mean and variance adjusted (WLSMV) estimation with probit links to account for the binary nature of both outcome variables. The pattern of results remained consistent when re-estimated using maximum likelihood with bootstrapped standard errors, treating the binary outcome variables as continuous. All analyses are robust to dropping covariates unless otherwise specified. See S1 Appendix for analyses without covariates. The results are shown in Table 2, which presents the primary paths of interest.

**Table 2:** SEM Path Coefficients for Contribution, Self-Efficacy, Social Responsibility, and Political Participation.

Path	*B*	SE	95% CI	*z*	*p*
Effects on Contribution					
Self-efficacy ➔ Contribution	0.07	0.02	[0.03, 0.11]	3.12	.002
Social responsibility ➔ Contribution	0.09	0.01	[0.07, 0.11]	8.50	<.001
Effects of Contribution on Political Participation					
Contribution ➔ Political donations	0.14	0.03	[0.09, 0.20]	4.99	<.001
Contribution ➔ Political volunteering	0.20	0.03	[0.14, 0.25]	6.67	<.001
Indirect Effects Through Contribution					
Self-efficacy ➔ Contribution ➔ Political donations	0.01	0.004	[0.002, 0.02]	2.64	.008
Social responsibility ➔ Contribution ➔ Political donations	0.01	0.003	[0.01, 0.02]	4.29	<.001
Self-efficacy ➔ Contribution ➔ Political volunteering	0.01	0.005	[0.004, 0.02]	2.82	.005
Social responsibility ➔ Contribution ➔ Political volunteering	0.02	0.003	[0.01, 0.03]	5.24	<.001

N = 2,680. Model controlled for age, gender, race and ethnicity (dichotomized), social class context (dichotomized), marital status (dichotomized), working status (dichotomized), life satisfaction, and psychological well-being.

#### Components of self-perceived social contribution.

First, we tested whether the theorized components of social contribution were indeed related to the construct. Self-efficacy (*B* = 0.07, *p* = .002) and social responsibility (*B* = 0.09, *p* < .001) were both significantly positively related to self-perceived social contribution.

#### Self-Perceived social contribution and political participation.

Second, we tested whether social contribution was related to two measures of political participation. The results show self-perceived social contribution was indeed significantly positively related to being more likely to donate to (*B* = 0.14, *p* < .001) and volunteer for (*B* = 0.20, *p* < .001) political causes.

#### Indirect effects of self-efficacy and social responsibility on political participation through contribution.

Third, we tested whether social contribution mediated the relationships between the component variables (i.e., self-efficacy and social responsibility) on measures of political participation (i.e., political donations, political volunteering), shown in Table 2. The results provided support that self-perceived social contribution significantly mediated the effect of self-efficacy on political donations (*B* = 0.01, *p* = .008) and political volunteering (*B* = 0.01, *p* = .005). The results also showed that self-perceived social contribution significantly mediated the effect of social responsibility on political donations (*B* = 0.01, *p* < .001) and political volunteering (*B* = 0.02, *p* < .001). Notably, while self-perceived social contribution mediated these relationships, the direct effects showed more complex associations: self-efficacy had a negative direct effect on political donations (**B* *= −0.10, *p* = .002) but no significant direct effect on volunteering (**B* *= −0.04, *p* = .294). Social responsibility showed a positive direct effect on donations (**B* *= 0.06, *p* < .001) and volunteering (**B* *= 0.09, *p* < .001), alongside the mediated pathways.

It is also possible that the components of social contribution (i.e., self-efficacy and social responsibility) have an interactive relationship with social contribution and political participation. We tested this possibility with an additional SEM model that included an interaction term for self-efficacy and social responsibility and report the results in S1 Appendix. While the interaction term did not reach standard levels of significance in the model, the effects trended to suggest the interaction between self-efficacy and social responsibility was positively related to perceived social contribution and had indirect effects on political donations and volunteering. Thus, it is possible that in addition to additive effects found in the primary SEM model, self-efficacy and social responsibility may also depend on one another when relating to perceived social contribution and political participation. However, we exert caution when interpreting these results given the lack of statistical significance (p-values ranging from 0.102–0.120). Regardless, both pattern of results suggest that the two components (i.e., self-efficacy and social responsibility) were positively related to perceived social contribution which in turn was associated with greater political participation.

We also explored the possibility that social contribution is a mechanism through which global well-being relates to political participation. To do so, we tested if social contribution mediates the effects of life satisfaction and psychological well-being on political participation. The results, which are reported in S1 Appendix, provide evidence that perceived social contribution fully mediated the effect of global well-being on political participation.

### Discussion

Study 2 found that self-perceived social contribution was significantly related to two additional measures of political participation (i.e., donating to and volunteering for political causes). This replicates the pattern of effects found in Study 1 in a large, representative sample from 1996. Importantly, Study 2 further revealed the relationship between contribution and political outcomes remained robust to controlling for global measures of well-being. Additionally, Study 2 confirmed that the theorized components of social contribution (i.e., self-efficacy and social responsibility) indeed underlie the construct in the context of modeling political outcomes.

## General discussion

Across two samples from two distinct points in the history of American politics and history, the current paper finds that feeling as though one contributes to society is meaningfully associated with greater political participation. Specifically, people who believe their life and daily activities provide something of value to society were more likely to intend to vote in a national election, be willing to engage in social activism, seek out rather than avoid information about the election, and donate to and volunteer for political causes. For a political system that requires active participation on the part of its citizens in order to function, these studies identify a novel aspect of well-being that is related to being politically engaged. Further, Study 2 provides empirical support for the theoretical claims that the perceived ability to achieve one’s goals and a sense of duty to a larger collective underly a feeling of being a worthy contributor to society which in turn is related to engaging in the political process. Thus, Study 2 provides evidence that self-efficacy and social responsibility, as previously theorized, are indeed related to perceived social contribution.

These studies add to the literature on how well-being is associated with political engagement and extend it by specifically identifying that self-perceived social contribution plays a critical role above and beyond measures of life satisfaction and psychological well-being which has traditionally been the focus of this literature [13,14,17]. The current studies also add to the literature on the implications of social well-being by finding that in addition to impacting physical health and psychological functioning [39,40], one’s sense of being a contributing member of the larger society has implications for being active and engaged in politics. This extension provides additional empirical evidence for the theorized importance of the collective nature of well-being and the consequences of feeling socially well (or not) within a broader community or society [22,36]. Finally, Study 2 also found additional empirical support for the theoretical assumption that self-efficacy and social responsibility underly a sense of contributing [22]. In doing so, Study 2 provided additional evidence that believing one can achieve their goals as well as feeling an obligation to the larger collective society are important components of feeling as though one contributes to society in the context of political outcomes.

In addition to the theoretical implications of this work, there are practical implications worth discussing as well. Some scholars have advised that politicians who seek reelection should have a vested interest in improving the well-being of their constituents, given that happy and well citizens are more likely to vote [13]. The current work provides additional support for this recommendation and suggests that politicians who wish to lead a collective that is politically engaged (via voting, donating, volunteering, and seeking information) may do well by paying particular attention to whether their constituents feel as though they are valued and that they contribute something worthwhile to their communities and society. Such work echoes past calls about the political consequences of people who feel left behind and as if they do not matter (e.g., [56,57]). While there are many open questions regarding how to best foster a feeling of being valued and an integral part of a collective, it may involve greater recognition and structural support for the many ways people already contribute (e.g., [58]). Relatedly, for organizations who aim to increase voter engagement and participation, the current work suggests that communication strategies that recognize the ways people are already contributing to society and are valued members of their community may be effective at motivating people to join their movement. Such work could complement past strategies for increasing political participation by focusing on people’s identity and self-perception as a voter (e.g., [59]) by focusing on people as “contributors” that deserve to have their voice heard.

Taken together, this work centers perceived social contribution as a critical measure of well-being that has a place in political participation processes. How people think and feel about themselves as meaningful contributors to society links to their engagement in fundamental democratic processes. Our findings support the theorized association between perceived social contribution and political participation, however, it is important to note that the relationship is likely bidirectional. Like the larger literature on well-being and political participation, political engagement likely reciprocally fosters a sense of contributing to society [13]. Further, we acknowledge that perceived social contribution is not the only concept of well-being that links to political participation, but it is one that predicts such participation above and beyond established global measures of psychological well-being. We provide evidence that perceived social contribution plays a key role and hope future work continues to investigate its effects to understand those who may (and may not) be willing or able to participate in pro-democratic processes.

### Limitations and future directions

These studies have important limitations worthy of further investigation in future research. First, like much of the literature on well-being and political engagement, the findings reflect correlational, not causal, relationships. Though correlational, the temporal nature of Study 1’s measures support the direction of feelings of contribution being associated with future political engagement (e.g., intending to vote, being willing to engaging in political activism, planning to seek out information about an election). However, it is possible that a third variable could confound the relationship between feelings of contribution and political engagement. For example, some of the characteristics of the U.S. electoral system (e.g., the need to register to vote) can vary by geographic location (e.g., different states have specific procedures, deadlines, etc.) and could potentially influence not only political participation but also societal integration and perceived social contribution. Individuals in states with greater access to voting may feel more connected to their communities and perceive themselves to be greater contributors to society. While our analyses control for key demographic factors and both studies feature national samples, future work could examine how structural and psychological correlates of political participation affect one another and whether the association between perceived social contribution and political participation varies across different electoral contexts. Further, while the models presented in the paper control for common demographic characteristics known to be related to perceived contribution and political engagement (e.g., social class, age, etc.; [3,36,48,49]), future work can investigate the associations found here against a more stringent set of alternative hypotheses and third variables. For example, future work could investigate the relationships found here while accounting for personality traits, such as extraversion, which has been shown to be associated with one’s sense of contributing as well as some forms of political participation [60,61].

Additionally, we note that in Study 1, the relationship between social contribution and willingness to engage in activism was not robust to controlling for political efficacy measures. This suggests that political efficacy may be an important mediating variable or confounder in this relationship, highlighting the need for future research to more carefully disentangle the roles of social contribution and political efficacy in predicting different forms of political participation. Additional research can also further investigate how self-efficacy and social responsibility are related to perceived social contribution. Study 2’s additive model found evidence for the two constructs being independent predictors. However, it is possible that self-efficacy and social responsibility interact such that feeling as though one is obligated to contribute may be more likely to promote political participation when one also feels as though they have the means to carry out their intentions. On the other side, feeling efficacious may more strongly relate to political participation when focused on one’s perceived personal responsibility to positively impact society. Such a possibility would complement past work showing efficacy and anger interact to predict political behavior [62,63]. The interactive model as reported in the Supplemental Information shows trends suggesting some evidence for an interactive relationship between self-efficacy and social responsibility. However, the model did not yield statistically significant results and therefore warrants caution when interpreting the results. Future work can specifically investigate the nuances in how self-efficacy and social responsibility relate to one another, perceived social contribution, and political engagement.

Future work can also leverage experimental designs to determine if a causal pathway exists between feeling as though one contributes to society and a readiness to participate politically. Such work could add to the literature on social well-being by determining how to shift a person’s global sense of whether they are contributing members of society. This potential future work would also be well-positioned to explore the possibility of creating an “upward spiral” or setting off a recursive process such that feeling as though one contributes to society causes people to engage in society through politics which then causes people to believe they are valued, contributing members of society [64,65].

The current set of studies considers a number of different behaviors related to participating in the political system ranging from seeking (and not avoiding) information about an election to giving one’s time and money to political causes to being willing to engaging in various forms of activism. This broad set of outcomes adds to the literature on how well-being is related to being engaged politically. However, while behavioral intentions are closely linked to engaging in the behavior itself [66,67], future work can aim to more closely capture behavior by asking for more specific evidence of engagement in political actions (e.g., asking about the date, time, and activity) or partnering with political organizations to see if any interventions might increase political engagement.

While the current studies control for demographic and political factors, they do not systematically investigate their potential effects on the relationship between feelings of contribution and political engagement. It is possible that believing one contributes meaningfully to society may be particularly impactful among those who are at risk of feeling excluded from the political process and could serve as an activating and empowering force. Further, a broad literature has established that liberals and conservatives tend to rely on varying moral foundations and cognitive processing strategies when making political judgments [68–70]. Some past work suggests liberals and conservatives differ in the expansiveness of their circle of moral concern, with liberals tending to cast a wider circle of concern (e.g., extending to the entire planet and beyond) in comparison to conservatives who focus their moral concern more closely within their immediate social networks (e.g., to close friends, family, and community members) [71]. Given this pattern, it is possible conservatives may identify more with aspects of their life that they believe contribute to a more localized community and therefore seek out political behaviors with more localized impact. In contrast, it is possible that liberals would be more likely to conceive of their contributions in terms of affecting a larger collective (e.g., the world) and seek out political behaviors that have the potential for broader impact. Thus, while Study 1 controlled for participant political party, future work can further investigate if one’s political ideologies affect how feeling as though one contributes to society is related to engaging in the political process.

## Conclusion

The consequences of feeling as though one is a valued member of society (or not) have been known to impact individual health and well-being. The current findings suggest that such feelings also affect the well-being of the collective and health of the democratic system, as those who believe they contribute to society are more likely to engage in the political process. If someone does not believe their life and daily activities provide something of value to society and are therefore also less likely to be engaged in that broader society politically, this experience may serve as another avenue in which people are further excluded and then even less likely to feel that they are a part of and can contribute to that broader collective. These results suggest that finding ways of affirming people as important, contributing members of society, based on whatever they currently do or by merely existing, could have critical consequences for the individual and well as the political system in which they inhabit.

## Supporting information

S1 Appendix Supporting information file. (DOCX)
