# Supplementary material for: Perceived social contribution and its associations with political participation
Source: PLoS One. 2025 Sep 5;20(9):e0330385. doi: 10.1371/journal.pone.0330385 (PMC12412917; doi:10.1371/journal.pone.0330385)
Supplement: S1 Appendix — (DOCX) [file pone.0330385.s001.docx]

**SUPPLEMENTAL INFORMATION**

**Perceived Social Contribution and its Associations with Political Participation**

**S1 Table. Social activism scale items and weights**

| Item | Item(s) that Weight is Based On | Weight |
| --- | --- | --- |
| Advocate for my candidate (e.g., wear a t-shirt, hat, bumper stickers, yard signs etc.) | Average of "Advocate for the issue on your person (e.g., wear a t-shirt, hat)" and "Advocate for the issue with physical promotion materials (e.g., bumper sticker, sign in yard)" | 56.5 |
| Volunteer for my candidate's campaign | "Become a volunteer for an organization" | 70 |
| Post about my candidate on social media | "Post about this issue on social media" | 59 |
| Talk about the candidates with someone who agrees with me | "Talk about the issue with someone who agrees with you about the issue" | 60 |
| Canvassing neighborhoods for my candidate | "Hand out flyers with information on the issue" | 62 |
| Attend a rally | "Attend a protest" | 63 |
| Educate myself on the candidates | "Educate yourself on this issue (e.g., read books, search online)" | 64 |
| Donate money | "Donate money to organization" | 63.5 |
| Educate myself on the issues (e.g., read book, search online) | "Educate yourself on this issue (e.g., read books, search online)" | 64 |
| Talk about the election with someone who views the candidates differently than I do | "Talk about the issue with someone who sees this issue differently than you do" | 65 |
| Educate others on the issues | "Educate others on the issue" | 66 |
| Attend a protest | "Attend a protest" | 63 |
| Become a volunteer for an organization that supports my views (e.g., "get out the vote", Planned Parenthood, National Rifle Association) | "Become a volunteer for an organization" | 70 |

*Note.* Items weights are taken from Turner et al., (2024).

**S2 Table.** **Additional study 1 sample demographics**

| Demographic | Response Options | Percent of Sample |
| --- | --- | --- |
| Gender | Man | 46.33 |
|  | Woman | 46.23 |
|  | Non-binary | 0.29 |
|  | Did not respond | 7.15 |
| Race and Ethnicity | White/European American | 64.16 |
|  | Black/African American | 12.11 |
|  | Latino/Latina | 8.48 |
|  | Asian/Asian American | 5.72 |
|  | Multiracial | 5.43 |
|  | Native American/Pacific Islander | 2.10 |
|  | Other race/ethnicity | 2.00 |
| Race Dichotomized | White | 64.16 |
|  | Asian, Asian American, Black, African American, Latino/Latina, Native American, Pacific Islander, multiracial or any other race/ethnicity listed | 35.84 |
| Education Dichotomized | Less than a four-year college degree | 63.78 |
|  | More than a four-year college degree | 36.22 |
| Marital Status Dichotomized | Currently married | 43.09 |
|  | Not currently married | 56.91 |
| Working Status Dichotomized | Currently working | 46.90 |
|  | Not currently working | 52.24 |
|  | Did not respond | 0.86 |
| Political Party | Democrat | 48.43 |
|  | Republican | 46.81 |
|  | Independent | 3.15 |
|  | Prefer not to say | 0.86 |
|  | Other (write in) | 0.76 |
| Political Party Dichotomized | Democrat | 50.81 |
|  | Republican | 48.14 |
|  | Did not respond | 1.05 |

*Note.* Participants selected all races or ethnicities that applied. Participants who selected more than one race/ethnicity were coded as multiracial. The marital status variable compares currently married versus single, divorced, or widowed participants. The currently working variable compares working (paid employee, self-employed) versus not working (temporary layoff from a job, looking for work, retired, disabled, or other). Participant age ranged from 18-90 years (*M* = 54.47, *SD* = 15.99).

**S3 Table. Study 1 results without covariates**

|  | *B* | *SE* | 95% CI | *z* or *t* | *p* | *R^2^* |
| --- | --- | --- | --- | --- | --- | --- |
| Voting Intention | 0.49 | 0.10 | [0.29, 0.70] | 4.70 | <.001 | .07 |
| Willingness to Engage in Social Activism | 0.09 | 0.02 | [0.05, 0.13] | 4.20 | <.001 | .02 |
| Information Seeking | 0.16 | 0.03 | [0.11, 0.20] | 6.14 | <.001 | .03 |
| Information Avoidance | -0.31 | 0.03 | [-0.36, -0.35] | -11.62 | <.001 | .11 |

*Note.* Voting intention reports Nagelkerke pseudo-R^2^ given the binominal distribution.

**S4 Table. Study 1 results controlling for internal and external political efficacy**

|  | *B* | *SE* | 95% CI | *z* or *t* | *p* | *R^2^* |
| --- | --- | --- | --- | --- | --- | --- |
| Voting Intention | 0.32 | 0.14 | [0.05, 0.60] | 2.34 | .019 | .38 |
| Willingness to Engage in Social Activism | 0.03 | 0.02 | [-0.02, 0.07] | 1.14 | .26 | .19 |
| Information Seeking | 0.09 | 0.03 | [0.04, 0.15] | 3.30 | .001 | .12 |
| Information Avoidance | -0.20 | 0.03 | [-0.36, -0.35] | -6.97 | <.001 | .21 |

*Note.* Voting intention reports Nagelkerke pseudo-R^2^ given the binominal distribution. In addition to controlling for internal political efficacy and external political efficacy, the models control for the following covariates: social class (dichotomized into with and without a four-year college degree), age, gender, race and ethnicity (dichotomized into participants who are White and participants of color), marital status (dichotomized into currently married and not currently married), working status (dichotomized into currently working and not currently working), and political party (dichotomized into Republican and Democrat). Internal political efficacy and external political efficacy scale items and details are reported in Study 1 materials.

**S5 Table. Additional study 2 sample demographics**

| Demographic | Response Options | Percent of Sample |
| --- | --- | --- |
| Gender | Woman | 50.71 |
|  | Man | 49.29 |
| Race and Ethnicity | White | 88.58 |
|  | Black and/or African American | 6.16 |
|  | Asian or Pacific Islander | 1.01 |
|  | Multiracial | 0.82 |
|  | Native American or Aleutian Islander/Eskimo | 0.78 |
|  | Other race/ethnicity | 2.65 |
| Race Dichotomized | White | 88.58 |
|  | Black, African American, Asian, Pacific Islander, Native American, Aleutian Islander, Eskimo, Multiracial, or any other race/ethnicity listed | 11.42 |
| Education Dichotomized | Less than a four-year college degree | 69.48 |
|  | More than a four-year college degree | 30.52 |
| Marital Status Dichotomized | Currently married | 64.81 |
|  | Not currently married | 35.19 |
| Working Status Dichotomized | Currently working | 62.46 |
|  | Not currently working | 37.54 |

*Note.* The marital status variable compares currently married versus single, divorced, or widowed participants. Participant age ranged from 20-74 years (*M* = 46.61, *SD* = 12.92).

**S6 Table. Study 2 results without social responsibility item measuring obligation to vote**

| Path | *B* | SE | 95% CI | *z* | *p* |
| --- | --- | --- | --- | --- | --- |
| Effects on Contribution | | | | | |
| Self-efficacy 🡪 Contribution | 0.06 | 0.02 | [0.02, 0.11] | 2.84 | .005 |
| Social responsibility 🡪 Contribution | 0.08 | 0.01 | [0.06, 0.10] | 7.21 | < .001 |
| Effects of Contribution on Political Participation | | | | | |
| Contribution 🡪 Political donations | 0.15 | 0.03 | [0.09, 0.20] | 5.19 | < .001 |
| Contribution 🡪 Political volunteering | 0.2 | 0.03 | [0.14, 0.26] | 6.76 | < .001 |
| Indirect Effects Through Contribution | | | | | |
| Self-efficacy 🡪 Contribution 🡪 Political donations | 0.01 | 0.004 | [0.002, 0.017] | 2.49 | .013 |
| Social responsibility 🡪 Contribution 🡪 Political donations | 0.01 | 0.003 | [0.006, 0.016] | 4.20 | < .001 |
| Self-efficacy 🡪 Contribution 🡪 Political volunteering | 0.01 | 0.005 | [0.003, 0.022] | 2.62 | .009 |
| Social responsibility 🡪 Contribution 🡪 Political volunteering | 0.02 | 0.003 | [0.009, 0.021] | 4.93 | < .001 |
| *Note.* N = 2,680. Model controlled for age, gender, race and ethnicity (dichotomized), social class context (dichotomized), marital status (dichotomized), working status (dichotomized), life satisfaction, and psychological wellbeing. The measure of social responsibility includes three of the four items used in the main text, removing the item assessing the obligation to “to vote in local and national elections.” | | | | | |

**S7 Table. Study 2 results without covariates**

| Path | *B* | SE | 95% CI | *z* | *p* |
| --- | --- | --- | --- | --- | --- |
| Effects on Contribution | | | | | |
| Self-efficacy 🡪 Contribution | 0.29 | 0.02 | [0.25, 0.133] | 13.67 | < .001 |
| Social responsibility 🡪 Contribution | 0.13 | 0.01 | [0.11, 0.15] | 11.01 | < .001 |
| Effects of Contribution on Political Participation | | | | | |
| Contribution 🡪 Political donations | 0.17 | 0.02 | [0.012, 0.22] | 6.96 | < .001 |
| Contribution 🡪 Political volunteering | 0.19 | 0.03 | [0.14, 0.24] | 7.36 | < .001 |
| Indirect Effects Through Contribution | | | | | |
| Self-efficacy 🡪 Contribution 🡪 Political donations | 0.05 | 0.01 | [0.03, 0.06] | 6.20 | < .001 |
| Social responsibility 🡪 Contribution 🡪 Political donations | 0.02 | 0.004 | [0.01, 0.03] | 5.87 | < .001 |
| Self-efficacy 🡪 Contribution 🡪 Political volunteering | 0.05 | 0.01 | [0.04, 0.07] | 6.48 | <.001 |
| Social responsibility 🡪 Contribution 🡪 Political volunteering | 0.02 | 0.004 | [0.02, 0.03] | 6.13 | < .001 |
| *Note.* N = 2,680. Model does not include any covariates (i.e., it does not control for demographic factors or global measures of well-being). | | | | | |

**S8 Table. Study 2 results with self-efficacy and social responsibility interaction term**

| Path | *B* | SE | 95% CI | *z* | *p* |  |
| --- | --- | --- | --- | --- | --- | --- |
| Effects on Contribution | | | | | | |
| Self-efficacy 🡪 Contribution | -0.06 | 0.08 | [-0.214, 0.101] | -0.71 | .48 |  |
| Social responsibility 🡪 Contribution | -0.002 | 0.06 | [-0.118, 0.114] | -0.03 | .973 |  |
| Self-efficacy x Social responsibility 🡪 Contribution | 0.02 | 0.01 | [-0.003, 0.035] | 1.63 | .102 |  |
| Effects of Contribution on Political Participation | | | | | | |
| Contribution 🡪 Political donations | 0.14 | 0.03 | [0.085, 0.195] | 4.99 | <.001 |  |
| Contribution 🡪 Political volunteering | 0.20 | 0.03 | [0.139, 0.255] | 6.69 | <.001 |  |
| Indirect Effects Through Contribution | | | | | | |
| Self-efficacy 🡪 Contribution 🡪 Political donations | -0.008 | 0.01 | [-0.030, 0.014] | -0.70 | .484 |  |
| Social responsibility 🡪 Contribution 🡪 Political donations | -0.0003 | 0.01 | [-0.016, 0.016] | -0.03 | .973 |  |
| Self-efficacy x Social responsibility 🡪 Contribution 🡪 Political donations | 0.002 | 0.001 | [-0.001, 0.005] | 1.55 | .120 |  |
| Self-efficacy 🡪 Contribution 🡪 Political volunteering | -0.011 | 0.02 | [-0.042, 0.020] | -0.70 | .482 |  |
| Social responsibility 🡪 Contribution 🡪 Political volunteering | -0.0004 | 0.01 | [-0.023, 0.022] | -0.03 | .973 |  |
| Self-efficacy x Social responsibility 🡪 Contribution 🡪 Political volunteering | 0.003 | 0.002 | [-0.001, 0.007] | 1.59 | .112 |  |
| *Note.* N = 2,680. Model controlled for age, gender, race and ethnicity (dichotomized), social class context (dichotomized), marital status (dichotomized), working status (dichotomized), life satisfaction, and psychological wellbeing. | | | | | | |

**S9 Table. Study 2 analysis of social contribution mediating the effect of global well-being on political participation**

| Path | *B* | SE | 95% CI | *z* | *p* |
| --- | --- | --- | --- | --- | --- |
| Global wellbeing 🡪 Contribution (*a*) | 0.62 | 0.03 | [0.56, 0.68] | 21.38 | < .001 |
| Contribution 🡪 Political participation, controlling for global well-being (*b*) | 0.18 | 0.03 | [0.13, 0.23] | 7.15 | < .001 |
| Global wellbeing 🡪 Political participation, controlling for contribution (*c'*) | 0.02 | 0.04 | [-0.06, 0.10] | 0.46 | .646 |
| Indirect effect | 0.11 | 0.02 | [0.08, 0.14] | 6.82 | < .001 |
| Total effect (*c*) | 0.13 | 0.04 | [0.06, 0.20] | 3.65 | < .001 |
| *Note.* N = 2,680. Latent variables: Global wellbeing (life satisfaction, psychological well-being), Political Participation (political giving, political volunteering). Model controlled for age, gender, race and ethnicity (dichotomized), social class context (dichotomized), marital status (dichotomized), working status (dichotomized), life satisfaction, and psychological wellbeing. | | | | | |
